# PROPAB: Computation of Propensities and Other Properties from Segments of 3D structure of Proteins

**DOI:** 10.6026/97320630014190

**Published:** 2018-05-31

**Authors:** Rifat Nawaz UL Islam, Chittran Roy, Parth Sarthi Sen Gupta, Shyamashree Banerjee, Debanjan Mitra, Sahini Banerjee, Amal Kumar Bandyopadhyay

**Affiliations:** 1 Department of Zoology, The University of Burdwan, West Bengal, 713104, India; 2 Department of Biotechnology, The University of Burdwan, West Bengal, 713104, India; 3 Department of Chemistry, IISER Berhampur, Berhampur, Odisha, 760010, India; 4 Department of Biotechnology, Institute of Genetic Engineering, West Bengal, 700128, India

**Keywords:** Protein, Chou and Fasman, propensity, properties, Program, secondary structure

## Abstract

Residues in allelic positions, in the local segment of aligned sequences of proteins show wide variations. Here, we describe PROPAB that computes the propensity tables for helix, strand and coil types from multiple 3D structure files following ab initio statistical procedure. It also classifies them in range specific and chain specific manners. It further computes percentage composition and physicochemical properties along with residues propensities. It also prepares FASTA files for different segments (helix, strand and coil) in the exact order that they follow in the sequence. Representative analyses on orthologous (homologous across species) proteins demonstrate wide segmental variations of physicochemical properties. Such variations provide insights to relate the adaptation of these proteins in a given functional constraint under diverse environmental conditions. Thus, the program finds applications in the structural and evolutionary analysis of proteins.

**Availability:**

PROPAB is freely available at http://sourceforge.net/projects/propab/for worldwide user.

## Background

Global minimal structure is spontaneously formed by amino acid sequence via intermediate levels of structures (such as helix, strand and coil), when kept under appropriate solution conditions [1]. As an intermediate structure drives forward the formation of tertiary structure, prediction of the earlier from amino acid sequence has been an ongoing effort. Starting from the elegant yet simplistic statistical method of Chou and Fasman [2], various other recent methods have been developed in last forty years to understand these codes of amino acid residues and eventually to predict secondary structures from a given sequence [3]. Due to reasonably high accuracy of Chou and Fasman method (>70%) [2, 3], which is almost equivalent to the most modern one [4], many popular web-server are using the earlier method for prediction of secondary structures [3]. At this point, it is worth noting that in these prediction methods, the level of inaccuracy (~25%) sought further developments and sometime older methods are fallen under criticism [3]. It would, however, be interesting to follow up the variability, the source of inaccuracy, in terms of (i) its distribution among different segment of secondary structures (helix, strand and coil), and (ii) the changes of amino acid propensity for functionally identical proteins operating under diverse environmental conditions (e.g. thermophilic, halophilic and mesophilic etc). Amino acid residues may have different physicochemical properties under different solvent conditions [5]. How are the properties of different segments of secondary structures of orthologous proteins affected? Would these variations be the source of inaccuracy in measured propensity? An efficient procedure would be useful that not only computes and classify amino acid propensities in error-free, user-friendly manner for any number of structures with any number of chains in them but also critically analyzes physicochemical properties of segments of helices, strands and coils by their self-extraction from structure files. Additionally these FASTA files could further be used for the analysis of variability, evolutionary properties [6], physicochemical and sequence properties [7, 8]. It is with this broad perspective in mind; we have developed PROPAB that not only implements the famous Chou and Fasman [2] method for propensity but also for the extraction of other above mentioned properties.

## Methodology

**Figure 1:**
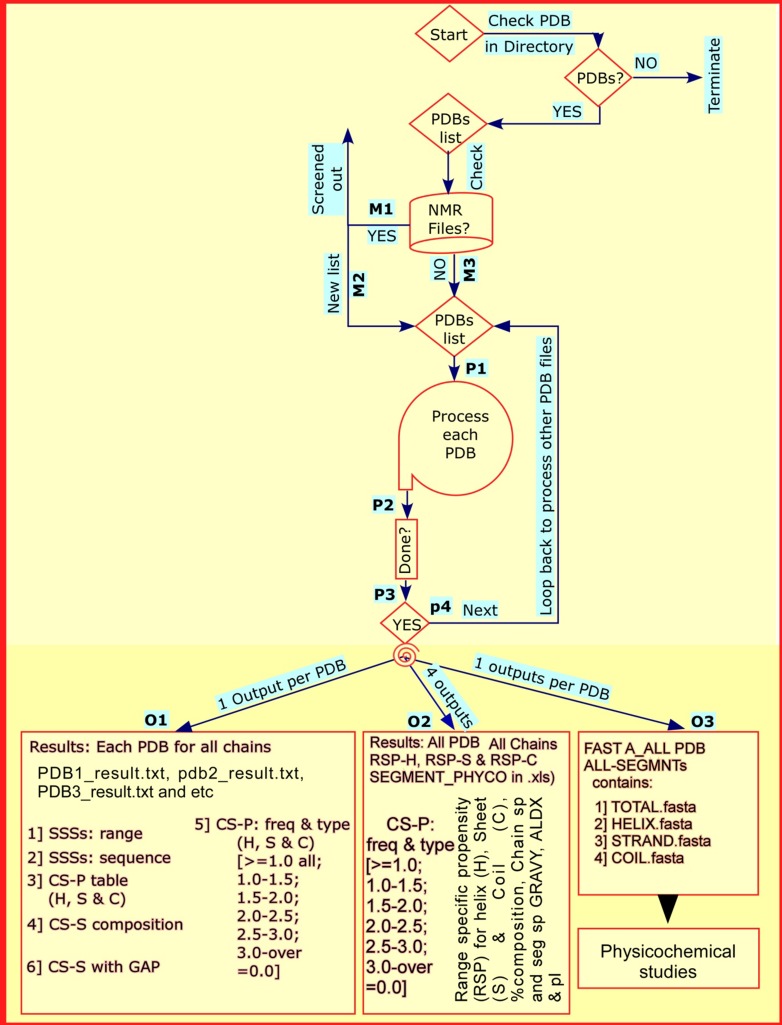
Flowchart for the functioning of the program PROPAB. Upon start of the program, it first checks for NMR files in the working directory. If these are present, they are screened out and then a list of X-ray structure files is made. Each PDB file is processed separately. Once completed, three kinds of outputs: O1 (six itemed one output per PDB), O2 (all chains, all PDB files specific outputs for Helix, Strand and Coil i.e. four outputs) and O3 (one output, FASTA files for Total, Helix, Strand and Coil containing sequences from all chains and all PDBs) are produced. PDB: protein Data Bank; SSSs: Secondary Structure Sequences; H: Helix; S: Strand; C: Coil; CS-S: Chain Specific Sequence; CS-P: Chain Specific Propensity; Freq: Frequency.

The operating principle and design of the program PROPAB, is shown in the flowchart (Figure 1). Upon start the program, it checks for PDB or ENT files in the working directory. If present, it prepares a list of PDB files, otherwise terminates. It then verifies the list for NMR files (Figure 1- M1). If present, these are screened out and a new list (Figure 1 - M2) is made, otherwise continue with the earlier list (Figure 1 - M3). Such a design is adapted from earlier works [7, 9]. Now the program enters into processing phase (Figure 1 - P1). At this stage, PROPAB makes thorough checking and correction for chain discontinuity, such that the entire topology is successfully scanned. The program then redirects three types of outputs (Figure 1 - O1, O2 and O3) upon completion of analysis (via P2 and P3) and loop back for processing the next PDB file in the list (Figure 1 - P4) and so on, until it exhausts all PDB files in the list. While one output with many items per PDB is designed in O1, the program redirects results of all PDBs (and all chains) in O2 and O3. Here the program follows the plan of separation of analytical results of helix, strand and coil segments of all PDBs (and all chains), which causes four and one outputs in O2 and O3 respectively.

### Program input

**Figure 2:**
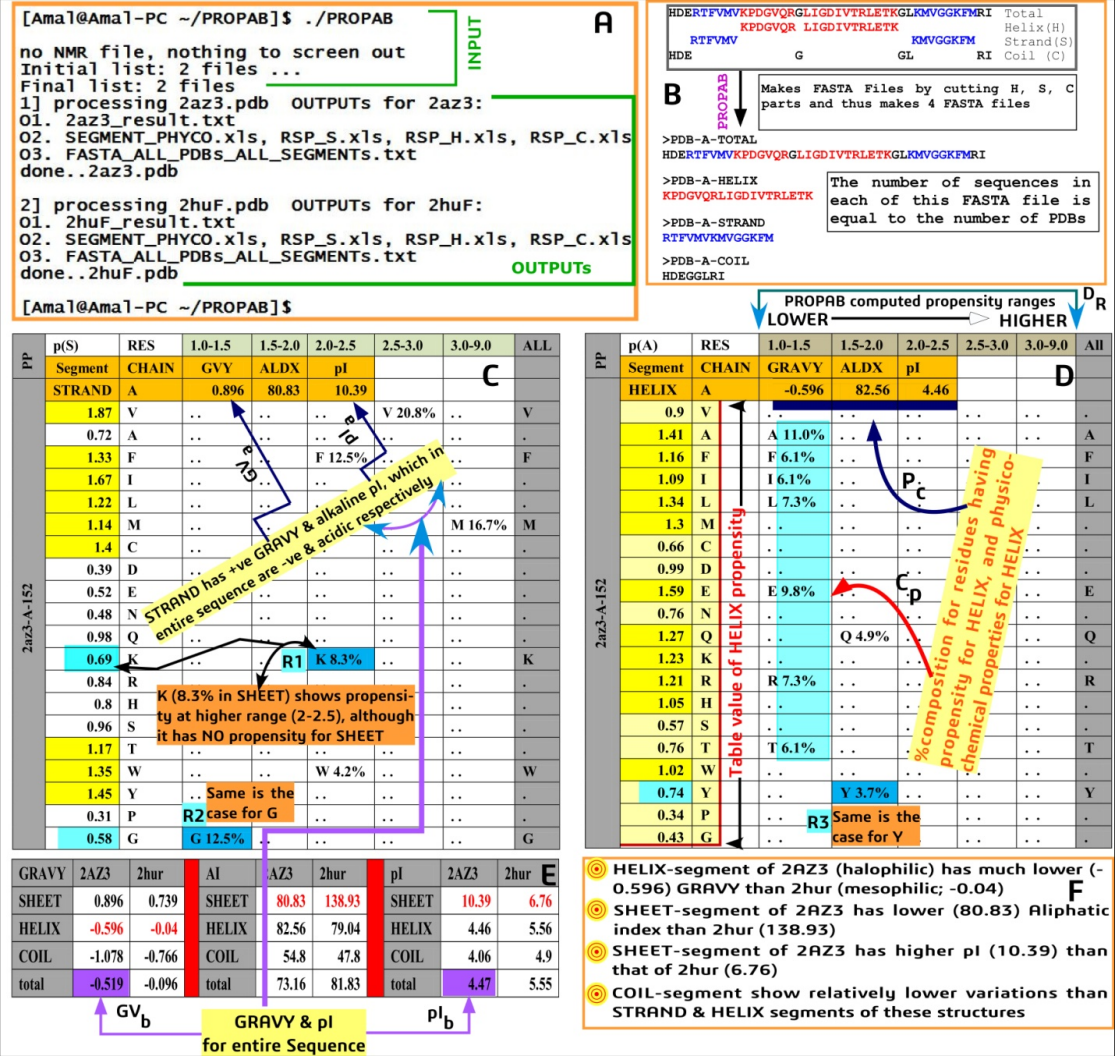
PROPAB extracted results show insightful observations. Running of PROPAB in CYGWIN 32 bit UNIX like environment, (A) that updates details of inputs and outputs in the screen. Program makes sequence from structure files (B) with GAP information, identify helix (red), strand (blue) and thus the Coil (black) regions (B). FASTA files are prepared for entire, helix, strand and coil sequences for all PDBs and all chains. Normalized composition (in %) and physicochemical properties are then computed, which are overcastted during the formation of range specific and chain specific propensity table for strand (C), helix (D) and coil (not shown) regions. Comparison of physicochemical properties of 2AZ3 (halophilic) and 2HUR (mesophilic) for different segments (E) shows that GRAVY for strand segment is positive (GVa) whereas it is negative for entire sequence (GVb). Similarly, pI for strand is much higher (pIa) than the entire protein (pIb; 2AZ3). Although known propensity is lower than unity, certain residues (e.g. R1, R2 for 2AZ3 and R3 for HUR2) show propensity at higher range with their normalized compositions for strand and helix segments. PROPAB presents segments (helix, strand, coil) propensity in range specific (DR) and chain specific manner (D), wherein %-compositions (CP) and physicochemical properties (PC) are also included.

The program requires crystallographic structure files as inputs in its working directory as earlier [7, 8]. It can process any number of structure files with any number of chains in them. Due to presence of variable number of models in NMR file, PROPAB avoids using NMR files as input [9], in that it efficiently screens them out, while preparing final processing list of structure files. These details are updated in the screen (Figure 2A).

### Program output

Three kinds of outputs (Figure 1 - O1, O2 and O3) are redirected in the working directory. O1 is PDB file specific output that contains six items (Figure 1 - O1). All PDBs (& all chains) specific four excel output (Figure 1 - O2) are redirected for range specific and chain specific propensity, %-composition and physicochemical properties. Finally, the program also produces a third output (O3) that contains FASTA files for complete, helix, strand and coil segments of structures in chain specific manner. This output may have far reaching application in terms of the estimation of variation in different segments of ensemble sequences. Figure 2 shows some of the interesting results in output as extracted by PROPAB, remarkable of which are

I. Preparation of FASTA files from structure files for different segments (Figure 2B),

II. Presentation of residue propensities in range specific (Figure 2 - DR), chain specific manner for different segments (Figure 2C for SHEET and Figure 2D for HELIX) of structures along with %-composition (Figure 2 - CP) and physicochemical properties (PC), along with inclusion of table values of propensities of residues that are worked out by Chou and Fasman [2].

The fact that the program PROPAB is capable of analyzing any number of structure files with any number of chains in them, appropriate selection of input structures (such as orthologous set that includes mesophilic, thermophilic and halophilic structures) and their analysis by the program seems to provide insightful results in output, especially in relation to segmental (helix, strand and coil) incorporation of variability in terms of propensity, composition and physicochemical properties, of which a glimpse is shown in output section (Figure 2C & Figure 2E).

### Caveats and future development

Program is written in AWK programming language, which can preferably run in any C shell UNIX prompt including CYGWIN 32 bit and also be made work in B shell LINUX and WINDOWS environment. Presently we are actively engaged in developing web interface to integrate SBION2 and ADSBET2 [9, 10] along with other related software tools of our laboratory [6, 7, 8, 11] such that their availability could reach to all academic users within an integrated web service.

## References

[1] Anfinsen CB. (1973). Science..

[2] Chou, Fasman. (1974). Biochemistry..

[3] Chen H (2006). BMC Bioinformatics..

[4] Montgomerie S (2006). BMC Bioinformatics..

[5] Wolfenden R (1981). Biochemistry..

[6] Gupta PS (2017). Bioinformation..

[7] Gupta PS (2014). Bioinformation..

[8] Banerjee S (2015). Bioinformation..

[9] Gupta PS (2015). Bioinformation..

[10] Nayek A (2015). Bioinformation..

[11] Nayek A (2015). Int. J Inst. Pharma. Life Sci..

